# A significant association between deltamethrin resistance, *Plasmodium falciparum* infection and the *Vgsc*-1014S resistance mutation in *Anopheles gambiae* highlights the epidemiological importance of resistance markers

**DOI:** 10.1186/s12936-016-1331-5

**Published:** 2016-05-23

**Authors:** Bilali Kabula, Patrick Tungu, Emily J. Rippon, Keith Steen, William Kisinza, Stephen Magesa, Franklin Mosha, Martin James Donnelly

**Affiliations:** Kilimanjaro Christian Medical University College (KCMUCo), Moshi, Tanzania; Amani Research Centre, National Institute for Medical Research, Muheza, Tanzania; Tukuyu Research Centre, National Institute for Medical Research, Tukuyu, Tanzania; Department of Vector Biology, Liverpool School of Tropical Medicine, Liverpool, UK; Global Health Division, RTI International, Dar es Salaam, Tanzania

**Keywords:** L1014S, kdr, *Vgsc*, *Anopheles gambiae*, *Plasmodium falciparum*, Tanzania

## Abstract

**Background:**

The success of malaria vector control is threatened by widespread pyrethroid insecticide resistance. However, the extent to which insecticide resistance impacts transmission is unclear. The objective of this study was to examine the association between the DDT/pyrethroid knockdown resistance mutation *Vgsc*-1014S, commonly termed *kdr,* and infection with *Plasmodium falciparum* sporozoites in *Anopheles gambiae*.

**Methods:**

WHO standard methods were used to characterize susceptibility of wild female mosquitoes to 0.05 % deltamethrin. PCR-based molecular diagnostics were used to identify mosquitoes to species and to genotype at the *Vgsc*-L1014S locus. ELISAs were used to detect the presence of *P.falciparum* sporozoites and for blood meal identification.

**Results:**

Anopheles mosquitoes were resistant to deltamethrin with mortality rates of 77.7 % [95 % CI 74.9–80.3 %]. Of 545 mosquitoes genotyped 96.5 % were *A. gambiaes.s*. and 3.5 % were *Anopheles arabiensis*. The *Vgsc*-1014S mutation was detected in both species. Both species were predominantly anthropophagic. In *A. gambiaes.s.*, *Vgsc*-L1014S genotype was significantly associated with deltamethrin resistance (χ2 = 11.2; p < 0.001). The *P. falciparum* sporozoite infection rate was 4.2 %. There was a significant association between the presence of sporozoites and *Vgsc*-L1014S genotype in *A. gambiaes.s.* (χ2 = 4.94; p = 0.026).

**Conclusions:**

One marker, *Vgsc*-1014S, was associated with insecticide resistance and *P. falciparum* infection in wild-caught mixed aged populations of *A. gambiaes.s.* thereby showing how resistance may directly impact transmission.

## Background

Malaria vector control in Africa relies on insecticide-treated nets (ITNs) and indoor residual spraying (IRS) which in turn depends on vector susceptibility. Great progress has been made during the past decade with the distribution of approximately 427 million ITNs, enough to cover over 80 % of the 840 million people at risk of malaria in sub-Saharan Africa [1]. Similarly, the number of people protected by IRS in the region increased from 13 million in 2005 to 81 million in 2010, accounting for approximately 11 % of the at risk population [1]. However, the widespread development of insecticide resistance in malaria vectors (reviewed in [2]) draws into question the sustainability of the gains achieved through insecticide-based malaria vector control.

Quantifying the insecticide susceptibility of vector populations is advocated as an integral part of malaria control campaigns [3]. Whilst there are numerous mortality and knockdown phenotyping assays there is only limited evidence of their associations with epidemiological outcomes; moreover they are subject to environment-induced error [4]. A number of researchers have advocated for the use of molecular diagnostics for resistance monitoring, as they should permit early detection of insecticide resistance. However, as discussed previously [5], there are barriers to the adoption of molecular markers by malaria control programmes for monitoring and evaluation beyond the obvious financial and logistical constraints. One of these major barriers is an observed variability in the power of molecular markers to predict results from phenotyping assays; partially a result of low power when markers approach fixation and/or the impact of environmental conditions on phenotype (discussed in [6]). Given the questionable epidemiological value of extant phenotyping tests, groups are developing improved assays [7]. However by associating known resistance markers directly with transmission indices (sporozoite infection) it is possible to remove the intermediate step of associating molecular resistance markers with an arbitrary resistance phenotype that may have no simple epidemiological interpretation.

## Methods

### Study site

The study was carried out in Zeneti village (5° 13′ S; 38° 39′ E) in Muheza district, northeast Tanzania. Malaria is intense and perennial with transmission peaking after the rainy season in May and June [8].

### Mosquito collections and processing

Mosquitoes were sampled between (November 2012 and May 2013). Indoor resting collections were used to obtain live females for deltamethrin susceptibility testing and pyrethrum spray catches (PSC) were used for mosquitoes that were collected for blood meal analysis. Collections were conducted between 06:00 and 09:00 h from 10–15 randomly selected houses. Live mosquitoes collected for susceptibility testing were provided with 10 % glucose solution and transported to the field insectary. Mosquitoes were sorted and morphologically identified to species. The blood meals from freshly fed females collected by PSC were squashed onto Whatman filter paper and dried. The remaining carcasses were stored individually over desiccant for laboratory processing.

### Insecticide susceptibility tests

Insecticide susceptibility tests were carried out using World Health Organization (WHO) tubes and standard operating procedures [9] with four replicates of 15–25 wild adult female mosquitoes per tube. Mosquitoes were exposed to papers impregnated with the WHO recommended discriminating concentration of 0.05 % deltamethrin prepared by University Sains Malasyia. Deltamethrin was chosen because it has a long history of use in the treatment of bed nets in the area, and *Anopheles gambiae s.l*. have developed resistance to the insecticide [10, 11]. The controls were exposed to paper impregnated with silicone oil. Mortality was scored after a 24-h holding period, during which time mosquitoes were provided with a 10 % sugar solution.

### Detection of *Plasmodium falciparum* infection and blood meal sources

*Plasmodium falciparum* sporozoite infections [12] and blood meal origins [13] were detected by ELISA (Enzyme Linked Immunosorbent Assay). Only the heads and thoraces were used to detect *P. falciparum* sporozoite infections. The blood ELISA screened for human, bovine and caprine antibodies.

### Molecular species identification and detection of point mutations in the voltage-gated sodium channel (Vgsc)

Molecular screening was conducted on mosquitoes collected for susceptibility tests/infection study and for detection of blood meal origin. The remaining body parts of these mosquitoes, not used in the ELISAs above, were transferred to a 96-well plate and DNA extracted using the DNeasy Blood and Tissue kit (Qiagen). The standard PCR protocol to identify *A. gambiaes.l*. species was used [14] together with a Taqman assay to screen for the 1014S mutation in the voltage-gated sodium channel [15].

### Ethical consideration

The study was approved by the Kilimanjaro Christian Medical College Research Ethics and Review Committee (CRERC) and the Medical Research Coordinating Committee (MRCC). The communities, where sampling was done, were informed on the project and informed consent sought from the local authorities within each community. Informed consent was also sought from the households where mosquito sampling was carried out.

## Results

A total of 948 adult female *A. gambiae* mosquitoes were exposed to the standard WHO dosage of deltamethrin; an additional 295 females were exposed to control papers in order to detect excess mortality due to exposure conditions. All control animals survived and are not considered further. Overall mortality rates of *Anopheles* mosquitoes 24 h post-exposure were 77.7 % (95 % CI 74.9–80.3 %). A total of 545 mosquitoes phenotyped by WHO tube test for deltamethrin susceptibility were successfully genotyped to species and for the *Vgsc*-L1014S point mutation. Out of these, 526 (96.5 %) were identified as *A. gambiaes.s*. and 19 (3.5 %) were *Anopheles arabiensis*. The *Vgsc*-1014S point mutation was detected in both *A. arabiensis* and *A. gambiaes.s.* The mutation was found at an allelic frequency of 0.45 (95 % CI 0.41–0.48) in *A. gambiaes.s.* and 0.32 (95 % CI 0.19–0.47) in *A. arabiensis.* The frequency of *Vgsc*-1014S in *A. arabiensis* and *A. gambiaes.s.* did not differ significantly (Fisher’s exact test p = 0.195) but given small number of *A. arabiensis* screened this analysis has limited power. Given their far larger sample size all subsequent analyses refer solely to *A. gambiaes.s*.. There was a significant association between deltamethrin resistance phenotype and *Vgsc*-L1014S genotype (Table 1).

**Table 1:** Associations between *Vgsc*-*L1014S* genotype and three phenotypes in *Anopheles gambiae s.s.* from Tanzania

Phenotype	N	*Vgsc*-*L1014S* genotype			Test statistic	p value
		Leu/Leu	Ser/Leu	Ser/Ser		
Deltamethrin resistant	127	31	55	41	O.R = 1.65^a^	p < 0.001
Deltamethrin susceptible	399	138	190	71	χ^2^ = 13.0^b^	p = 0.002
Percentage resistant		18.1 %	22.4 %	36.6 %	χ^2^ = 11.2^c^	P < 0.001
*P. falciparum* positive	22	4	9	9	O.R = 2.03^a^	p = 0.029
*P. falciparum* negative	504	165	236	103	Exact^d^	p = 0.072
Percentage *Pf.* positive		2.4 %	3.7 %	8.0 %	χ^2^ = 4.94^c^	p = 0.026
*Human* positive	355	94	189	72	O.R = 1.26^a^	p = 0.599
*Bovine* positive	17	6	8	3	Exact^d^	p = 0.756
Percentage human positive		94 %	95.9 %	96.0 %	χ^2^ = 1.60^c^	p = 0.205

Leucine (Leu) is the wildtype allele and Serine (Ser) the mutated resistance associated allele

^a^Odds ratio effect size was calculated based upon allelic data

^b^Test statistic and *p* value from a χ^2^ based on genotype frequencies

^c^Test statistic and p value from a χ^2^ test of trend

^d^A Fisher’s exact test was applied as one or more expected values <5

Of 526 *A. gambiaes.s*. mosquitoes for which a deltamethrin susceptibility phenotype and a *Vgsc*-*L*1014S genotype were obtained, 22 (4.2 %) were *P. falciparum* sporozoite positive. Despite this relatively low overall infection rate, there were a significantly higher proportion of sporozoite positives in *Vgsc*-1014S homozygotes relative to wildtype (Table 1). Heterozygotes showed an intermediate sporozoite infection rate relative to wild type and *kdr* homozygotes suggesting that the *kdr* allele may not be not fully recessive for the infection phenotype (Table 1).

Blood meal analyses of the mosquitoes sampled using PSC revealed high degrees of anthropophagy (Total n = 575; *A. gambiae* s.s. 95.4 %, *A. arabiensis* 71.3 %) although as expected *A. gambiae s.s.* had a significantly higher proportion of human blood meals (χ^2^ = 34.11; p < 0.001). Anthropophagy in *A. gambiae s.s.* was not associated with the presence of the *Vgsc*-1014S allele (Table 1).

## Discussion

The basis for selecting this geographical location were previous records of high levels of pyrethroid resistance [16]. Resistance may have remained high in this area due to the cumulative impact of continued ITNs use and scale-up. Insecticide resistance associated with scaling up of ITN and IRS interventions has been reported from across sub-Saharan Africa [17–20].

This study which, unusually, used female *A. gambiae s.s*. mosquitoes of mixed age, demonstrated a significant association between deltamethrin resistance phenotype and *Vgsc*-L1014S genotype. Studies based upon heterologous expression of the *Drosophila melanogaster Vgsc* in *Xenopus* eggs suggest that the 1014S mutation is maximally effective against, and may have been selected by, DDT [21]. Therefore, perhaps unsurprisingly, field studies showing an association between resistance to class II pyrethroids and 1014S genotype are few [6, 11]. In some instances this may, as noted in western Tanzania, reflect that the 1014S allele is close to fixation [22]. However in another study in neighbouring Uganda no genotype:phenotype association was observed even with the marker at an intermediate frequency and where associations were demonstrated for DDT and Class I pyrethroids [6, 23]. Given that additional resistance associated variants may accumulate on a *kdr* background [24] it may be that 1014S is a marker for two different haplotypes with differing resistance profiles, in Uganda/western Tanzania and eastern Tanzania (this study).

A key component of the entomological inoculation rate is the proportion of mosquitoes that are infected. There was a significant association between *Vgsc*-L1014S genotype and infection with *P. falciparum* in *A. gambiae s.s*. with infection rates in *Vgsc*-1014S homozygotes over three time higher that in wildtype females. The overall *P. falciparum* infection rate of 4.2 % was lower than that previously recorded in this area 7 % [25]. This may in part reflect a reduction in infection rates resulting from sub-lethal insecticide exposure. In a recent experimental study from Uganda sub-lethal exposure to deltamethrin resulted in reductions in both the proportion and intensity of infection of *P. falciparum* infection in *A. gambiae* [26]. However, in this study *Vgsc*-1014S homozygotes had sporozoite rates of 8 % suggesting that the mutation may in essence counteract the effects of sub-lethal exposure on infection.

For both deltamethrin susceptibility and sporozoite infection *Vgsc*-L1014S heterozygotes exhibit phenotypes that were intermediate between the extremes observed in both homozygote groups. This implies that for these phenotypes the *Vgsc*-1014S allele is not fully recessive as modelling studies on the *Vgsc*-1014F allele have previously suggested [27].

It was not possible resolve whether the association between infection and *Vgsc*-1014S genotype is a result of an increase in daily survival which means *Vgsc*-1014S carriers are more likely to survive the parasite extrinsic incubation period or a more direct *Vgsc*-1014S—*P. falciparum* interaction. Experimental studies may support this latter hypothesis as, in the absence of insecticidal pressure, higher sporozoite rates were recorded in *Vgsc*-1014F carrying *An. gambiae s.s.* compared to wild type [28]. RNAi silencing of a serine protease, ClipC9, in linkage disequilibrium with *Vgsc*-1014F resulted in reduced *P. falciparum* infection, suggesting a possible mechanism [29].

## Conclusions

This study demonstrated that in a Tanzanian population of *A. gambiae s.s*., phenotypic resistance to deltamethrin and infection with *P. falciparum* is significantly associated with the *Vgsc*-1014S point mutation. Whilst, fortunately there is no evidence of catastrophic failure of vector control programmes associated with the presence of this or other *Vgsc* mutations, these data suggest that in areas with high IRS and/or LLINs coverage *Vgsc*-1014S is potentially impacting control efforts by increasing *P. falciparum* infection rates. It is hoped that these data are a spur to malaria control programmes to integrate molecular resistance monitoring into their monitoring and evaluation programmes.
